# Strain-Dependent Lactic Acid Fermentation of *Capsosiphon fulvescens* Hydrolysate by *Lactobacillus* spp.

**DOI:** 10.3390/microorganisms13102295

**Published:** 2025-10-02

**Authors:** Hyeongjin Hwang

**Affiliations:** Department of Chemical and Biological Engineering, Chungwoon University, 113, Sukgol-ro, Michuhol-gu, Incheon 22100, Republic of Korea; hjhwang@chungwoon.ac.kr

**Keywords:** *Capsosiphon fulvescens*, seaweed hydrolysate, lactic acid fermentation, *Lactobacillus*, dilute-acid hydrolysis, sugar utilization

## Abstract

Seaweeds are promising third-generation biomass for biobased chemicals, yet their use for lactic acid (LA) production remains underexplored. We evaluated LA production from the dilute-acid hydrolysate of the aquacultured green alga *Capsosiphon fulvescens* (C.Agardh) Setchell & N.L. Gardner. The dried biomass contained 53.4% carbohydrate (dry-weight basis). HPLC showed a monosaccharide profile enriched in L-rhamnose and D-xylose, with lower levels of D-mannose, D-glucose, D-glucuronolactone, and D-glucuronic acid. Batch fermentations with three *Lactobacillus* strains revealed clear strain-dependent kinetics and carbon partitioning. Maximum LA titers/yields (time at maximum) were 2.0 g L^−1^/0.49 g g^−1^ at 9 h for *L. rhamnosus*, 2.3 g L^−1^/0.30 g g^−1^ at 36 h for *L. casei*, and 2.8 g L^−1^/0.23 g g^−1^ at 48 h for *L. brevis; L. rhamnosus* achieved the highest yield on sugars consumed, whereas *L. brevis* reached the highest titer by utilizing a broader sugar spectrum, notably xylose; *L. casei* showed intermediate performance with limited xylose use. Co-products included acetic and succinic acids (major) and trace 1,2-propanediol and acetaldehyde, consistent with flux through Embden–Meyerhof–Parnas versus phosphoketolase pathways. These results demonstrate that *C. fulvescens* hydrolysate is a viable marine feedstock for LA production and highlight practical levers—expanding pentose/uronic-acid catabolism in high-yield strains and tuning pretreatment severity—to further improve both yield and titer.

## 1. Introduction

Lactic acid (LA) has received considerable attention as a precursor of poly(lactic acid) (PLA), a well-known biodegradable plastic [1]. LA exists in two enantiomeric forms, L-lactic acid and D-lactic acid, and can be produced by either chemical synthesis or microbial fermentation. Chemical synthesis typically produces a racemic mixture (DL-lactic acid), which makes it difficult to control PLA’s physical properties. By contrast, microbial fermentation can produce optically pure L- or D-LA depending on the strain and substrate, enabling better control of polymer properties and access to high-molar-mass PLA [2,3]. The biotechnological production of lactic acid also offers advantages such as low-cost substrates, low process temperatures, and reduced energy consumption; as a result, LA production for PLA synthesis has increasingly trended toward microbial fermentation [4].

The biomass feedstock is a critical factor for biobased polymer and chemical production, often representing the largest portion (about 50%) of total cost [5]. Broadly, biomass feedstocks have been divided into first- and second-generation classes. The first generation involves food crops such as corn and sugarcane, whereas the second generation consists of lignocellulosics containing lignin, cellulose, and hemicelluloses. Recently, seaweed has emerged as a third-generation biomass. Seaweed has almost no lignin and can be easily pretreated for saccharification when compared with land plants such as corn stovers and wood [6,7,8]. In addition, compared with terrestrial plants, seaweed grows fast, resulting in high productivity per farming area. Unlike land plants, seaweed is free from the issues associated with utilizing water supplies when cultivated on a large scale. However, despite these advantages, there have been very few studies on the use of seaweeds as a biomass feedstock for the production of biobased products.

At the same time, seaweed hydrolysates present distinct bioconversion considerations. After acid pretreatment, green seaweeds often yield monosaccharide mixtures enriched in L-rhamnose and D-xylose, with mannose, glucose, and uronic acids as minors. Pentoses such as xylose are more susceptible than hexoses to acid-catalyzed degradation, making pretreatment severity a key lever to balance sugar release and degradation [8]. Moreover, many *Lactobacillus* strains lack complete xylose catabolic functions (e.g., xylose isomerase/kinase and related regulators), which can limit LA yields on pentose-rich substrates [9,10]. From a pathway perspective, hexoses typically flow through the Embden–Meyerhof–Parnas route, whereas pentoses are catabolized via the phosphoketolase pathway, which partitions carbon to LA and co-products such as acetate, with consequences for overall yield [11].

In this work, *Capsosiphon fulvescens* (*C. fulvescens*), a green seaweed commercially produced through aquaculture in Korea, was used as a biomass resource to produce lactic acid, and patterns of lactic acid fermentation were evaluated for three different *Lactobacillus* strains (*L. rhamnosus*, *L. casei*, and *L. brevis*). These comparisons are intended to clarify strain-dependent kinetics, titers, and yields on seaweed-derived sugars and to inform strain selection and pretreatment strategies for marine-biomass-to-LA processes.

## 2. Materials and Methods

### 2.1. Proximate Composition Analysis

*Capsosiphon fulvescens* was aquacultured by Sea & Tidal Co. (Jangheung, Jeollanam-do, Korea). Proximate composition was determined following the Korea Food Code methods [12]. Moisture was measured by the air-oven method; protein was calculated as total nitrogen × 6.25 using the Kjeldahl procedure [13]; ash was determined gravimetrically after incineration; lipid was quantified by ether extraction; carbohydrate was calculated by difference as 100 − (moisture + protein + ash + lipid).

Food-grade *C. fulvescens* (Ulvophyceae) is a siphonous, filamentous green seaweed whose cell-wall polysaccharides are rich in sulfated rhamnans, consistent with the rhamnose-enriched sugar profile observed after acid hydrolysis (Section 3.2) and the elevated ash/mineral contents in Table 1 [14,15]. No selective breeding or laboratory domestication was applied; aquaculture biomass was used as received.

### 2.2. Ultimate Elemental Analysis

CHONSP (carbon, hydrogen, oxygen, nitrogen, sulfur, phosphorus) analysis was performed on an elemental analyzer (Thermo Finnigan EA1108, Fisons Instruments, Somerset, NJ, USA). Samples were combusted at 1000 °C with standard catalysts/absorbents (WO_3_/Cu; nickel-plated carbon, nickel wool, quartz turnings, soda lime; magnesium perchlorate for moisture).

### 2.3. Mineral Analysis

Minerals were quantified by ICP-OES (Optima 7300 DV; PerkinElmer, Waltham, MA, USA). Samples were digested in HNO_3_/H_2_O_2_ at 150 °C for 2 h, then at 250 °C for 5 h using a digestion system (DigiPREP HT 250; SCP SCIENCE, Baie-D’Urfé, QC, Canada). Instrumental blanks were subtracted to correct background.

### 2.4. Acid Hydrolysis

Acid hydrolysis used sulfuric acid as catalyst. *C. fulvescens* (100 g) was mixed with 1 L of 0.5 M H_2_SO_4_ and hydrolyzed at 120 °C for 2 h (autoclave). The hydrolysate was neutralized with CaCO_3_, clarified by centrifugation, and filtered through a 0.22 μm syringe filter. The liquor was then analyzed by HPLC (below).

### 2.5. Lactic Acid Fermentation

*Lactobacillus rhamnosus* (KCTC 3237), *Lactobacillus casei* (KCTC 3260), and *Lactobacillus brevis* (KCTC 3498) were used. Pre-cultures were grown in basal medium plus glucose (20 g L^−1^). The basal medium (per liter) contained: peptone 10 g, beef extract 10 g, yeast extract 5 g, K_2_HPO_4_ 2 g, sodium acetate 5 g, tri-ammonium citrate 2 g, MgSO_4_·7H_2_O 0.2 g, MnSO_4_·4H_2_O 0.2 g, Tween 80 1 mL. For fermentations, seaweed hydrolysate was added to the basal medium and incubated in 200 mL baffled flasks (working volume 100 mL). Initial pH was 6.5. Conditions: 30 °C for *L. casei* and *L. brevis*, or 37 °C for *L. rhamnosus*; shaking at 170 rpm.

### 2.6. Analytical Methods

Organic acids (lactic, acetic) and total sugars were quantified by HPLC with refractive index detection. For organic acids, an Aminex HPX-87H column (Bio-Rad Laboratories, Hercules, CA, USA) operated at 35 °C with 5 mM H_2_SO_4_ as mobile phase (0.6 mL min^−1^). D-mannose and D-xylose were measured on a COSMOSIL Sugar-D column (Nacalai Tesque Inc., Kyoto, Japan) at 30 °C using 80% (*v*/*v*) acetonitrile in water (1.0 mL min^−1^). Samples were centrifuge-clarified and 0.22 μm-filtered prior to injection. Cell growth was monitored at OD_600_ with a UV–Vis spectrophotometer (Shimadzu UV-1700; Shimadzu Corporation, Kyoto, Japan).

### 2.7. Quantification and Calculations

Compound concentrations were determined by external calibration with authentic standards. LA yield on sugars consumed was calculated as in Equation (1).

$$Y_{LA/s}\;(g\;g^{-1})=\frac{C_{LA}-C_{LA,0}}{C_{S,0}-{\;C}_{S,t}}$$

where *C_LA_* and *C_LA__,__0_* are LA at time *t* and at *t* = 0, and *C_S,0_* and *C_S,t_* are total sugars at *t* = 0 and *t* (all in g L^−1^). “Total sugars” denotes the sum of quantified monosaccharides.

## 3. Results

### 3.1. Composition of Capsosiphon fulvescens

As shown in Table 1, the *C. fulvescens* used in this study comprised carbohydrate, protein, ash, and lipid, with carbohydrate as the largest fraction (53.4%), followed by protein (24.2%), ash (22.0%), and lipid (0.3%). The carbohydrate level is comparable to other green seaweeds such as *Ulva pertusa* (52.3%) and *Enteromorpha prolifera* (51.8%) [16,17], supporting the suitability of green seaweeds as carbon sources for fermentation. Mineral analysis indicated K, Na, Mg, and Ca as major components, with Al, Fe, Sr, and Cu in trace amounts (Table 1). The relatively high sulfur in the ultimate analysis is consistent with sulfated polysaccharides reported for *C. fulvescens* [14,15]. Although outside the scope of the present work, such compositional features suggest potential co-product opportunities (e.g., mineral or polysaccharide recovery) that could improve overall process economics if integrated with fermentation.

### 3.2. Hydrolysate Sugars and Yields; Comparison to Ulva Pertusa

As summarized in Table 2, sulfuric-acid hydrolysis produced a liquor containing L-rhamnose, D-xylose, D-mannose, D-glucose, D-glucuronolactone, and D-glucuronic acid. On a per-100 g dry biomass basis, the principal sugars were L-rhamnose (9.8 g) and D-xylose (7.6 g), followed by D-mannose (3.5 g), D-glucose (1.8 g), D-glucuronolactone (1.0 g), and D-glucuronic acid (0.3 g). Total quantified monosaccharides thus amounted to ~24.1%. The detection of D-glucuronolactone likely reflects in-process lactonization of D-glucuronic acid; at elevated pH (pH > 9) the lactone can revert to the acid. Compared with *U. pertusa* (30.1% total sugars [16]), the lower overall sugar yield for *C. fulvescens* (24.1%) may be related to the greater susceptibility of pentoses—especially xylose—to acid-catalyzed degradation [18]. Consistently, the D-xylose/D-glucose ratio in the *C. fulvescens* hydrolysate was high (7.6/1.8 ≈ 4.2 g g^−1^), markedly exceeding that of *U. pertusa* (0.625 g g^−1^), indicating a rhamnose/xylose-rich substrate that places specific demands on downstream fermentation.

### 3.3. Fermentation Performance Across Strains

**Figure 1:**
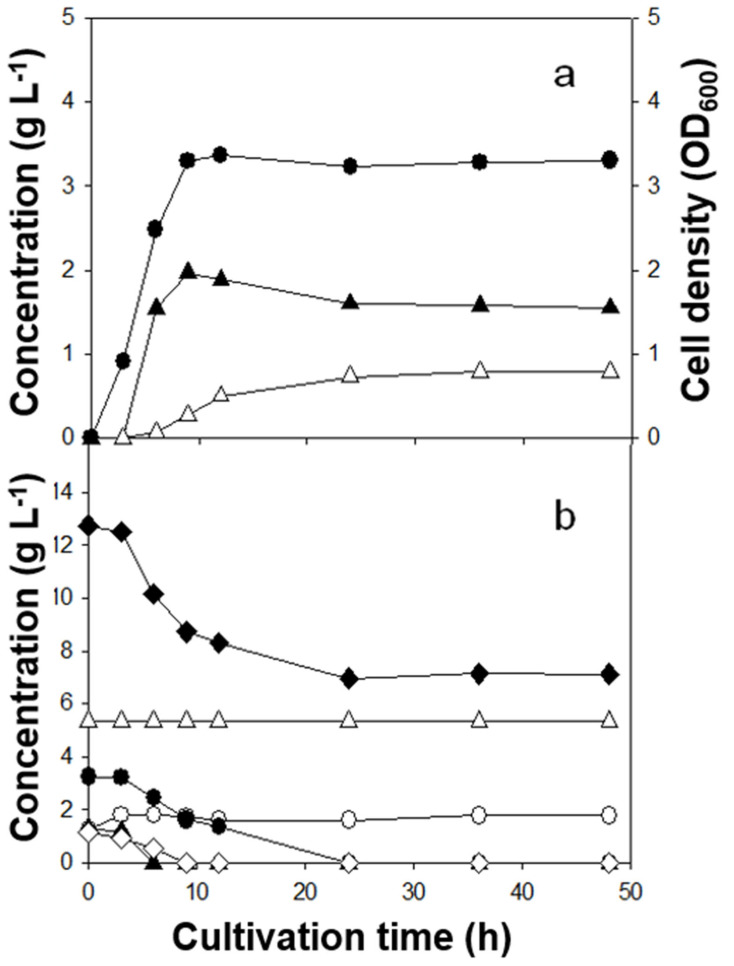
Lactic acid fermentation of *C. fulvescens* hydrolysate by *L. rhamnosus.* (**a**) Cell growth and product formation. ●, Cell density (OD_600_); ▲, lactic acid; △, acetic acid. (**b**) Sugar consumption profile. ◆, total sugar consumption; △, D- xylose; ●, L-rhamnose; ▲, D-glucose; ◇, D-mannose; ○, D-glucuronic acid.

**Figure 2:**
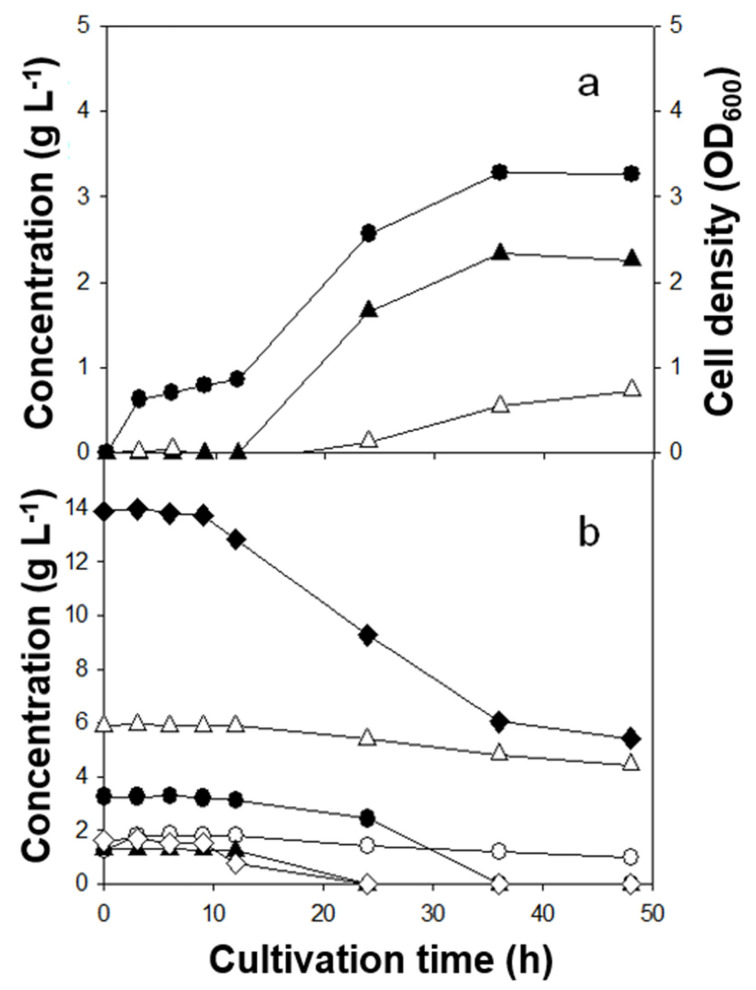
Lactic acid fermentation of *C. fulvescens* hydrolysate by *L. casei.* (**a**) Cell growth and product formation. ●, cell density (OD_600_); ▲, lactic acid; △, acetic acid. (**b**) Sugar consumption profile. ◆, total sugar consumption; △, D- xylose; ●, L-rhamnose; ▲, D-glucose; ◇, D-mannose; ○, D-glucuronic acid.

**Figure 3:**
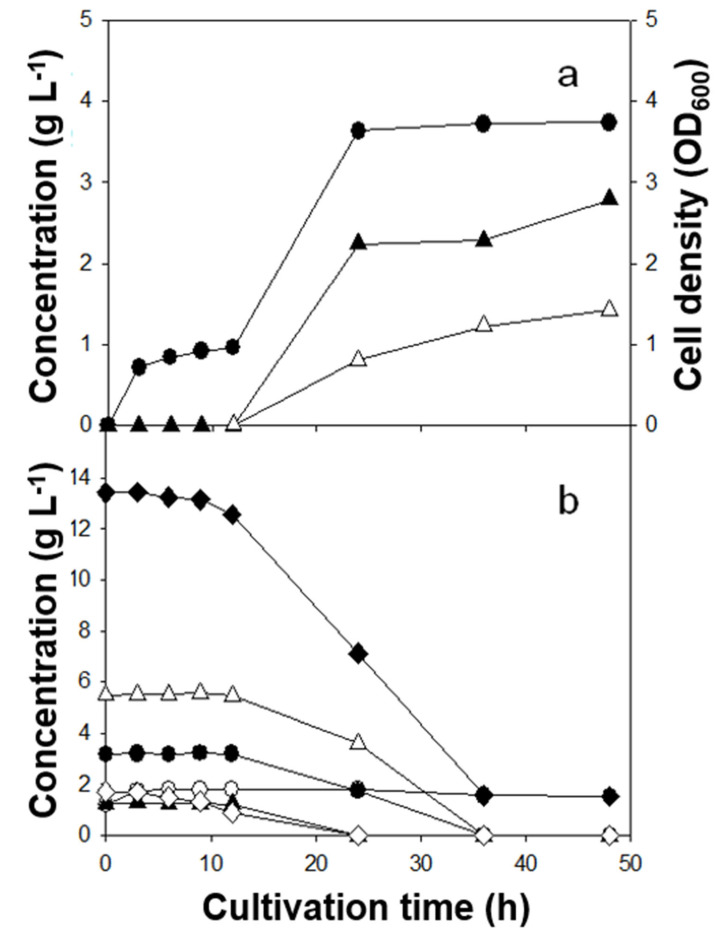
Lactic acid fermentation of *C. fulvescens* hydrolysate by *L. brevis*. (**a**) Cell growth and product formation. ●, Cell density (OD_600_); ▲, lactic acid; △, acetic acid. (**b**) Sugar consumption profile. ◆, total sugar consumption; △, D- xylose; ●, L-rhamnose; ▲, D-glucose; ◇, D-mannose; ○, D-glucuronic acid.

Batch fermentations with three Lactobacillus strains revealed clear strain-dependent behavior (Table 2; Figure 1, Figure 2 and Figure 3). Lactic acid (LA) yields on sugars consumed ranked *L. rhamnosus* (0.49 g g^−1^) > *L. casei* (0.30 g g^−1^) > *L. brevis* (0.23 g g^−1^). In contrast, the highest LA titer was achieved by *L. brevis* (2.8 g L^−1^), followed by *L. casei* (2.3 g L^−1^) and *L. rhamnosus* (2.0 g L^−1^). Kinetically, *L. rhamnosus* reached its maximum titer within ~9 h (short-cycle productivity), whereas *L. casei* and *L. brevis* peaked later (36 h and 48 h, respectively). In addition to LA, acetic and succinic acids were the major co-products; 1,2-propanediol and acetaldehyde were detected at trace levels. Because microorganisms can form 1,2-propanediol either via L-rhamnose catabolism or secondary conversion from LA [19], the small quantities observed here are consistent with limited flux through those routes under the present conditions. The ratio of total measured products to total sugars consumed was close to unity for *L. rhamnosus*, and <1 for *L. casei* and *L. brevis*, suggesting greater allocation to biomass and/or unmeasured pools at the sampling points for the latter strains.

### 3.4. Time-Course Profiles and Substrate Preferences

Time-course profiles (Figure 1, Figure 2 and Figure 3) clarified substrate preferences and growth–production coupling. *L. rhamnosus* reached maximal cell density rapidly (~9 h; Figure 1a) and consumed nearly all sugars except D-xylose and D-glucuronic acid (Figure 1b), indicating preference for D-glucose and L-rhamnose. In contrast, *L. casei* (36 h) and *L. brevis* (48 h) displayed slower trajectories, with limited sugar use by 9 h and substantial consumption after ~24 h. The inferred order of preference was D-mannose ≳ D-glucose > L-rhamnose > D-xylose > D-glucuronic acid for *L. casei*. *L. brevis* was similar except that it consumed almost all D-xylose. Limited xylose utilization by *L. rhamnosus* and *L. casei* is consistent with reports that many Lactobacillus strains lack complete xylose-catabolic machinery (xylose isomerase/kinase and related regulators) [9,10]. Cell density increased in parallel with LA formation across strains (Figure 1, Figure 2 and Figure 3).

## 4. Discussion

### 4.1. Pathway-Based Interpretation of Product Spectra

The observed product spectrum aligns with expected central-carbon routes. Hexoses (e.g., glucose, mannose) funneled through the Embden–Meyerhof–Parnas (EMP) pathway can, in principle, yield 2 mol LA per mol hexose, favoring high LA yield. Pentoses (e.g., xylose) channeled via the phosphoketolase (PK) pathway generate 1 mol LA + 1 mol acetate [11], inherently lowering LA yield and elevating acetate formation. Thus, the combination in *L. brevis* of higher titer (broader access including xylose) but lower yield (PK-associated acetate co-production) is mechanistically consistent. A notable feature was the post-peak decline in LA for *L. rhamnosus* with a concomitant increase in acetate between 9 and 48 h (Figure 1a), consistent with reports that LA can be converted to acetate and minor products under certain anaerobic conditions [20]. By contrast, LA did not decrease for *L. casei* and *L. brevis* over 48 h; continued sugar utilization coincided with gradual increases in both LA and acetate (Figure 2 and Figure 3), indicating that net production outpaced any secondary LA consumption in those cases.

Under prolonged anaerobic residence or low-pH, LA can be secondarily converted to other products by distinct guilds: heterofermentative LAB (e.g., *Lactobacillus buchneri*) oxidize LA to acetate with concomitant 1,2-propanediol; propionigenic bacteria convert lactate to propionate via acrylate or succinate routes; and chain-elongating consortia use lactate in reverse β-oxidation to produce C4–C8 carboxylates (e.g., butyrate, caproate). These routes explain the post-peak LA decrease with acetate rise observed for *L. rhamnosus* here and underscore standard control levers—earlier harvest, pH control/neutralization, and avoiding extended stationary phases—to minimize secondary LA consumption [20,21,22].

### 4.2. Process Implications and Improvement Levers

Cell density increased in parallel with LA formation across strains, indicating a strong coupling between growth and product accumulation under the present batch conditions. Practically, this suggests that recovery strategies timed near the end of the exponential phase could mitigate product-inhibition effects and potential secondary conversions (e.g., LA to acetate), particularly for *L. rhamnosus*. More broadly, two levers emerge for improving outcomes on seaweed-derived feedstocks: (i) enabling pentose/uronic-acid catabolism in high-yield strains (e.g., via introduction of xylose isomerase/kinase systems) to combine *rhamnosus*-like yields with broader substrate access.

Recent advances in lactobacilli engineering—including CRISPR/Cas multilocus editing, xylA/xylB knock-ins, and relief of carbon catabolite repression (CCR)—together with omics-guided identification of transporters/regulators for xylose/XOS uptake, PK-branching, and acid tolerance now offer practical routes to co-consume pentoses at high yield [23,24,25,26,27]. These developments support equipping high-yield strains (e.g., *L. rhamnosus*) with robust xylose/uronate catabolism while tuning pretreatment and pH control to boost both yield and titer.

(ii) Tuning acid-pretreatment severity to balance sugar release against pentose degradation. Together with pH management during fermentation, such strategies should increase both yield and titer while aligning strain capabilities with the characteristic rhamnose/xylose enrichment of *C. fulvescens* hydrolysates. In *Ulva* fermentations, co-cultivation, stepwise pH control, and customized pretreatment/enzymatic saccharification improved LA yield and productivity; recent syntheses also outline actionable levers such as buffering and nutrient supplementation [28,29,30]. These advances align with our observations and support a combined strategy of strain tuning plus pretreatment/pH control.

Lactic acid (LA) is a weak acid (pK_a ≈ 3.86), and the undissociated fraction increases as pH drops. At the final pH values measured here (4.5–5.0), approximately 7–19% of LA is undissociated, corresponding to ~1.7–5.8 mM at the observed titers (2.0–2.8 g L^−1^). Undissociated LA is membrane-permeant and can impair sugar uptake and growth in lactic acid bacteria, which is consistent with the late-stage slowdowns (Figure 1, Figure 2 and Figure 3) and the post-peak LA decrease observed for *L. rhamnosus*. In practice, standard mitigations include pH-stat neutralization (e.g., CaCO_3_ or NaOH), increased buffering, fed-batch sugar delivery, and in situ LA removal (e.g., electrodialysis or reactive extraction), all of which help sustain mixed-sugar uptake while limiting product inhibition.

### 4.3. Preliminary Energy and Economic Considerations

Two factors appear to dominate cost and energy demand. (i) Neutralization and salt management: 0.5 M H_2_SO_4_ pretreatment followed by CaCO_3_ neutralization generates solid salts (gypsum) and consumes base, increasing materials handling and disposal costs. Using milder acids, switching to NaOH/NH_4_OH-based pH-stat control during fermentation, or reducing the extent of external neutralization can mitigate salt loads. (ii) Downstream recovery at low titers: the maximum lactic acid (LA) titers observed here (2.0–2.8 g L^−1^) imply a high energy requirement per kg LA for concentration and purification; raising titers via pH control, fed-batch sugar delivery, and in situ LA removal (e.g., electrodialysis or reactive extraction) is therefore important. On the positive side, the low-temperature hydrolysis used here (120 °C, 2 h) has a comparatively modest steam demand relative to many lignocellulosic pretreatments, and integrated co-product recovery (e.g., minerals/polysaccharides noted in Section 3.1) could partially offset operating costs.

## 5. Conclusions

This study confirms that dilute-acid hydrolysate of the aquacultured green alga *Capsosiphon fulvescens* is a viable substrate for LA production. Its rhamnose/xylose-rich sugar profile strongly shaped fermentation behavior and led to clear strain-dependent outcomes: *L. rhamnosus* delivered the highest yield (0.49 g g^−1^), *L. brevis* achieved the highest titer by utilizing xylose (2.8 g L^−1^), and *L. casei* showed intermediate performance. Co-product patterns were consistent with hexose routing via the EMP pathway versus pentose routing via the phosphoketolase pathway, explaining the observed yield–titer trade-off. Practically, optimizing strain–substrate matching, pretreatment severity, and fermentation control (e.g., pH and recovery timing) are the key levers to improve both yield and titer on seaweed-derived hydrolysates.

## Figures and Tables

**Table 1:** Proximate, elemental (CHONSP), and mineral composition of *Capsosiphon fulvescens* (dry-weight basis).

Proximate Composition (g/100 g Dry Weight)		Ultimate Analysis (g/100 g Dry Weight)		Mineral Analysis (μg/g Dry Weight)		Sugar Composition of Acid Hydrolysate (g/100 g Dry Weight)	
Carbohydrate	53.4	C	34.81	K	36,310	D-xylose	15.0
Protein	24.2	H	5.28	Na	27,220	L-rhamnose	8.7
Lipid	0.3	O	43.85	Mg	7810	D-mannose	3.6
Ash	22.0	N	3.57	Ca	4810	D-glucose	1.8
		S	2.82	Al	1390	D-glucuronolactone	1.0
		P	0.32	Fe	1270	D-glucuronic acid	0.3
		others	9.35	Sr	50		
				Cu	10		
Total	100		100		78,870		30.4

**Table 2:** Summary of lactic acid fermentation of *C. fulvescens* hydrolysate.

Parameter	*L. rhamnosus*	*L. casei*	*L. brevis*
Cell density (OD_600_) ^a^	3.29	3.28	3.74
Initial pH	6.5	6.5	6.5
Final pH	4.8	5.0	4.5
Total product (g L^−1^) ^a^	3.9	4.6	5.0
Lactic acid (g L^−1^)	2.0	2.3	2.8
Acetic acid (g L^−1^)	0.3	0.5	1.4
Succinic acid (g L^−1^)	1.1	1.3	0
1,2-propanediol (g L^−1^)	0	0.2	0.2
Acetaldehyde (g L^−1^)	0.5	0.3	0.6
Total sugar consumed (g ^−1^) ^a^	4.0	7.8	11.9
L-rhamnose	1.6	3.2	3.2
D-xylose	0	1.1	5.5
D-mannose	1.2	1.6	1.7
D-glucose	1.2	1.3	1.3
D-glucuronic acid	0.1	0.6	0.2
D-glucuronolactone	0	0	0
Lactic acid yield (g/g) ^a^	0.49	0.30	0.23
Acetic acid yield (g/g) ^a^	0.07	0.07	0.12

^a^ Data for maximum cell density and lactic acid production which occur at fermentation time of 9 h for *L. rhamnosus*, 36 h for *L. casei*, and 48 h for *L. brevis*, respectively.

## Data Availability

The original contributions presented in this study are included in the article. Further inquiries can be directed to the author.
